# Supplementary figures and images for: Initiation of HIV-1 Gag lattice assembly is required for recognition of the viral genome packaging signal
Source: eLife. 2023 Jan 23;12:e83548. doi: 10.7554/eLife.83548 (PMC9908077; doi:10.7554/eLife.83548)

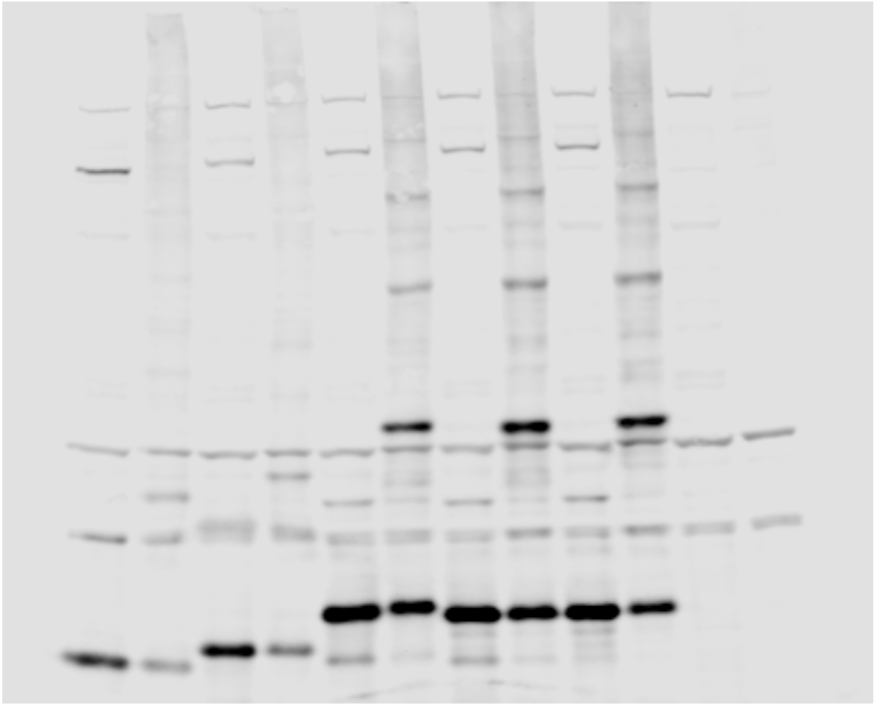

Supplement: Figure 2—source data 1. [file elife-83548-fig2-data1.zip › Figure 2 Source data 1/Fig 2C unlabelled.png]

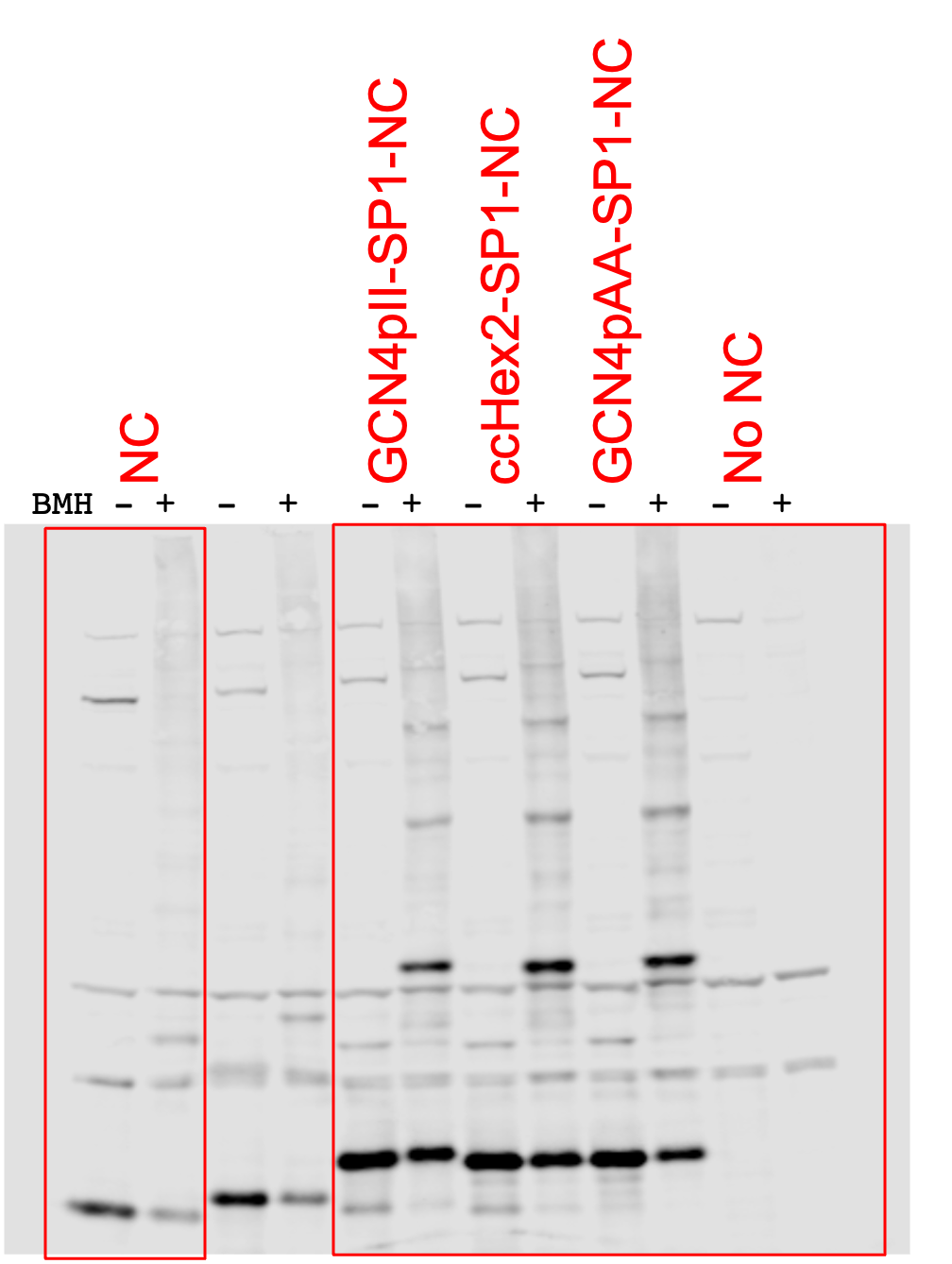

Supplement: Figure 2—source data 1. [file elife-83548-fig2-data1.zip › Figure 2 Source data 1/Fig 2C.png]

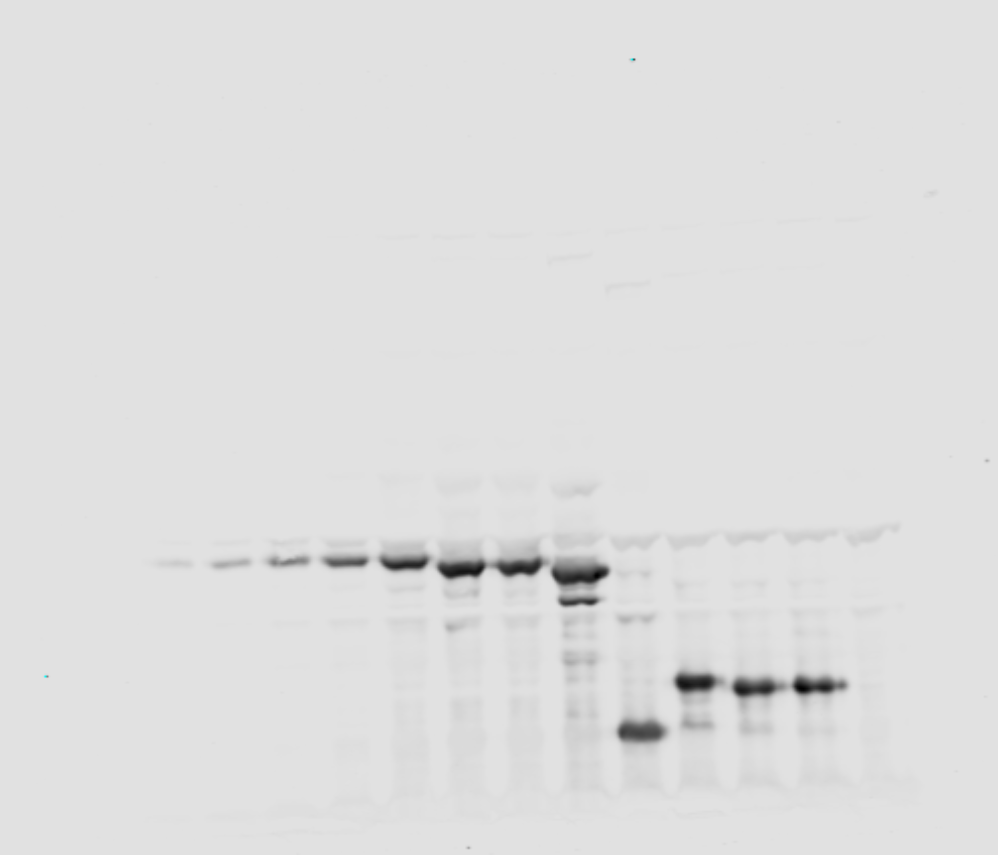

Supplement: Figure 2—figure supplement 2—source data 1. [file elife-83548-fig2-figsupp2-data1.zip › Figure 2-figure supplement 2 source data 1/Figure 2-figure supplement 2 upper unlabelled.png]

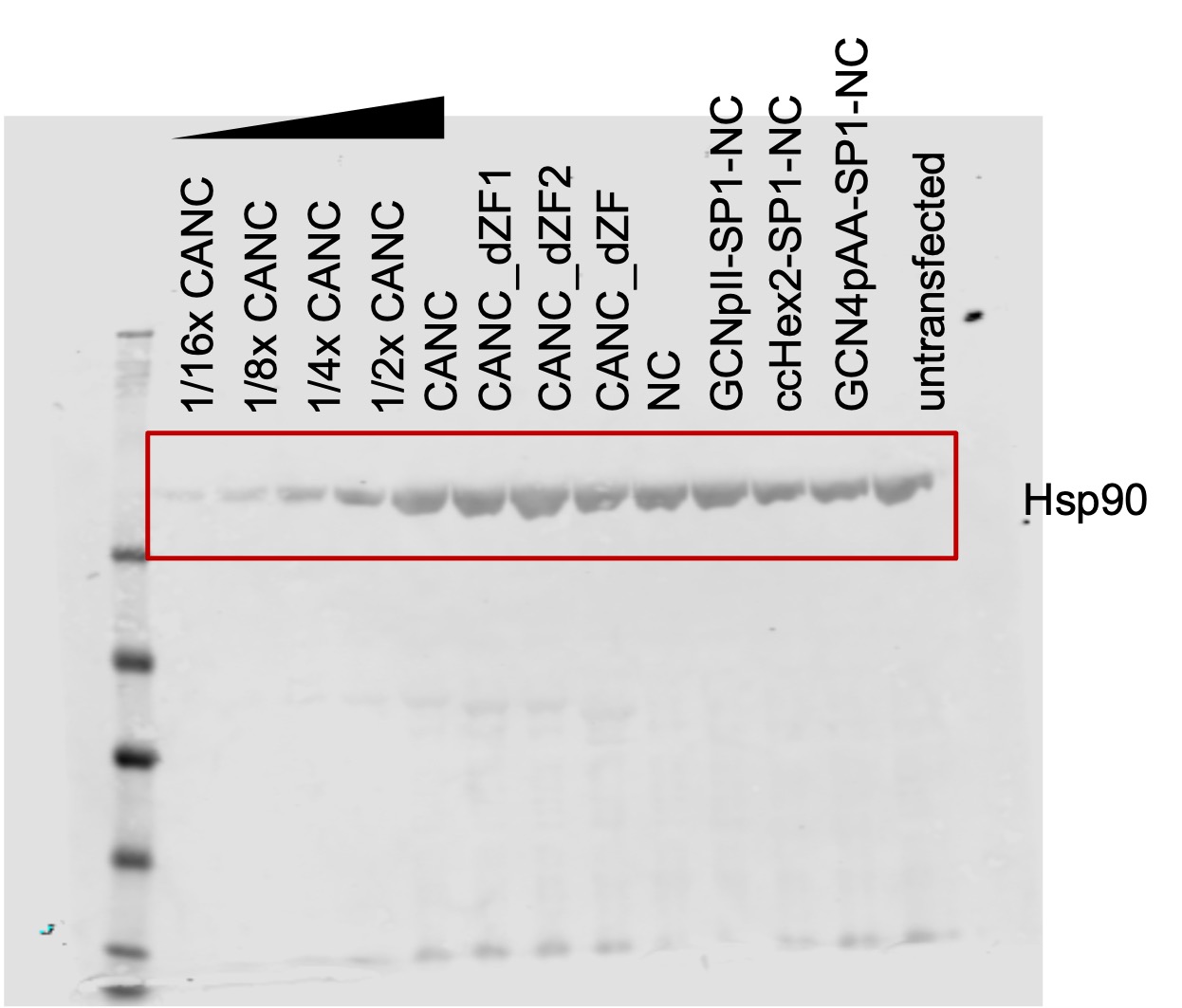

Supplement: Figure 2—figure supplement 2—source data 1. [file elife-83548-fig2-figsupp2-data1.zip › Figure 2-figure supplement 2 source data 1/Figure 2-figure supplement 2 lower.jpg]

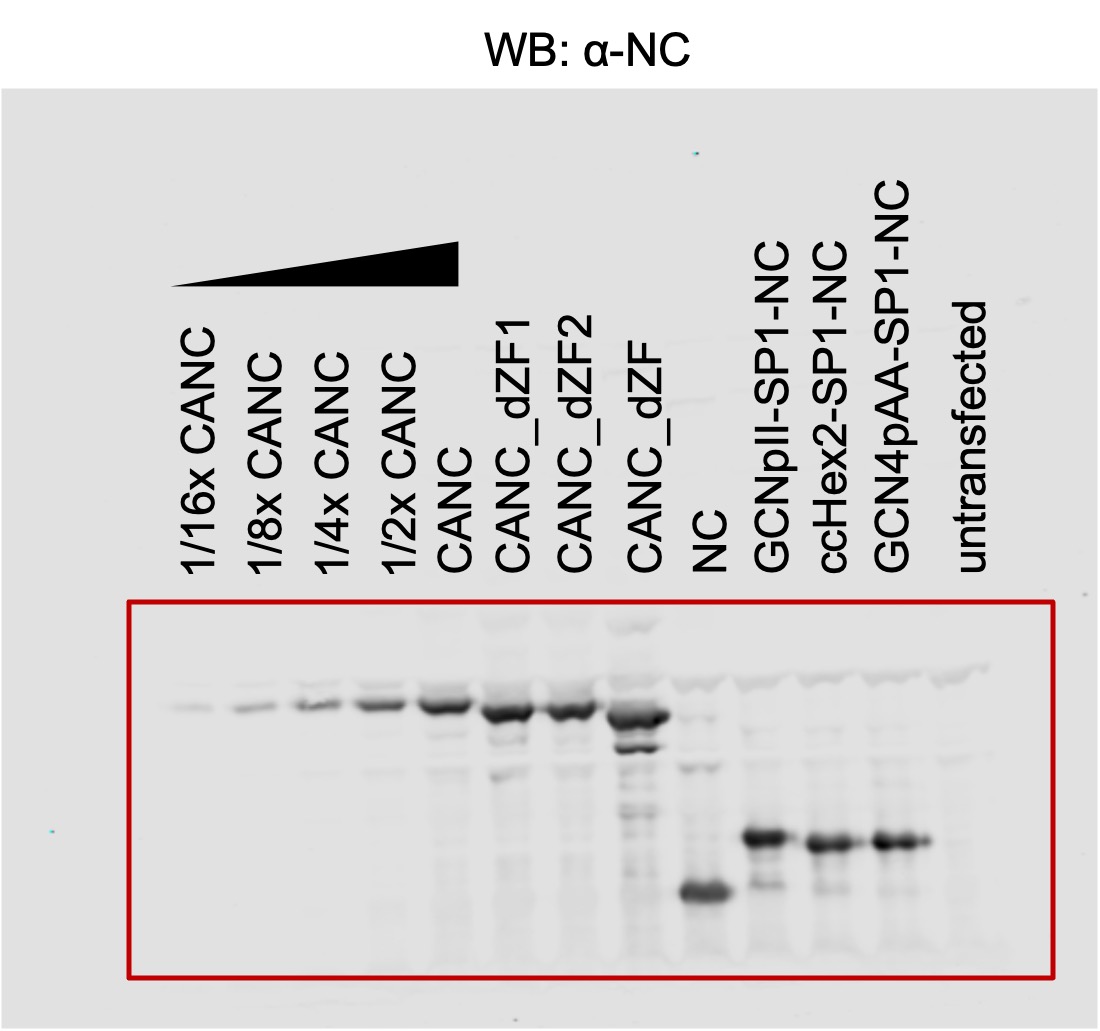

Supplement: Figure 2—figure supplement 2—source data 1. [file elife-83548-fig2-figsupp2-data1.zip › Figure 2-figure supplement 2 source data 1/Figure 2-figure supplement 2 upper jpg.jpg]

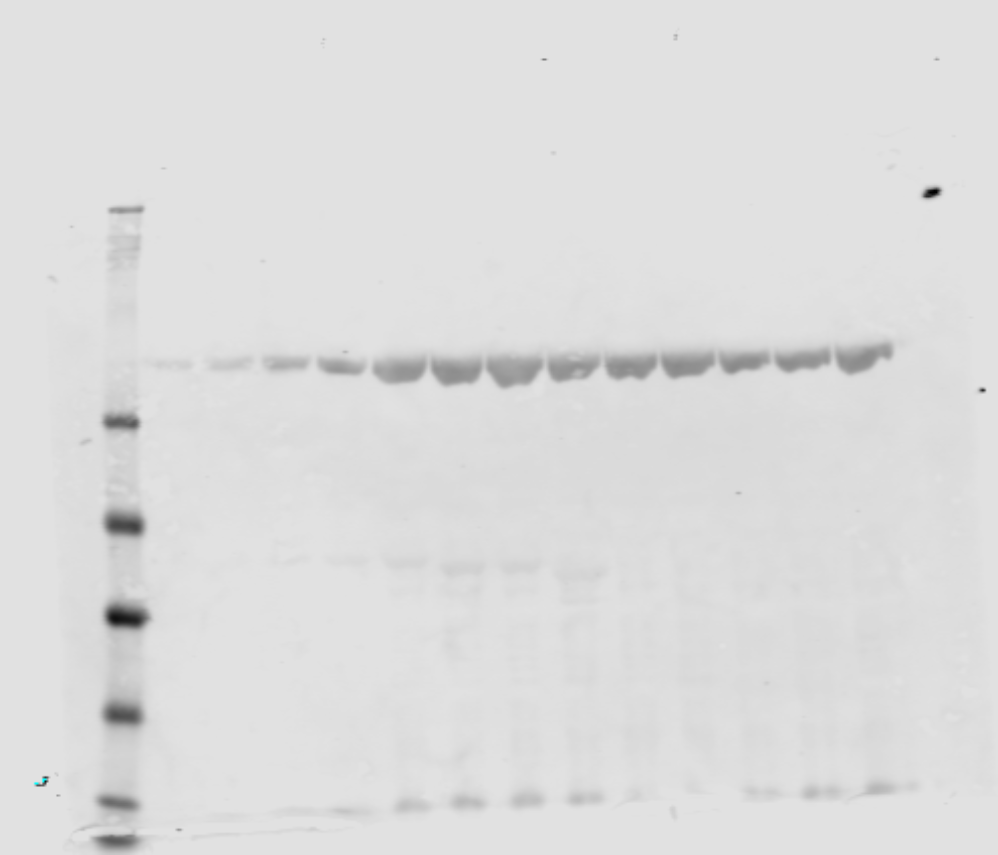

Supplement: Figure 2—figure supplement 2—source data 1. [file elife-83548-fig2-figsupp2-data1.zip › Figure 2-figure supplement 2 source data 1/Figure 2-figure supplement 2 lower unlabelled.png]

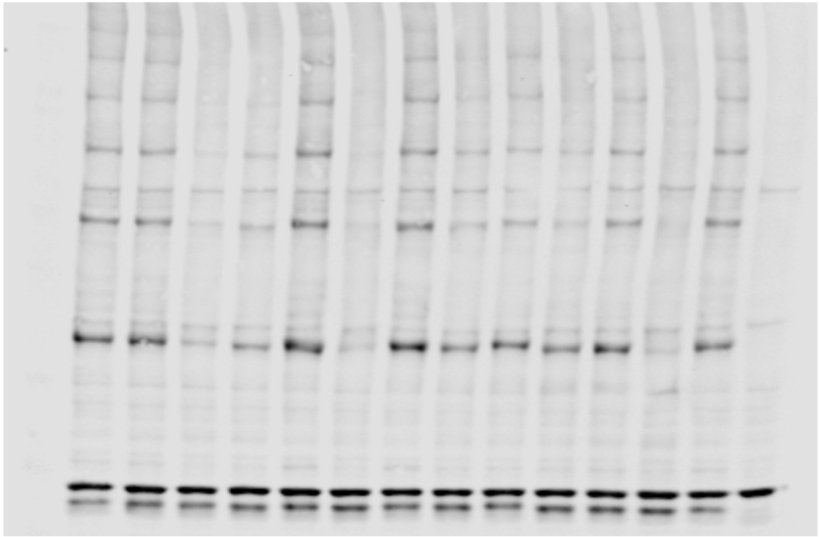

Supplement: Figure 4—source data 1. [file elife-83548-fig4-data1.zip › Figure 4 Source data 1/Fig 4A left upper unlabelled.png]

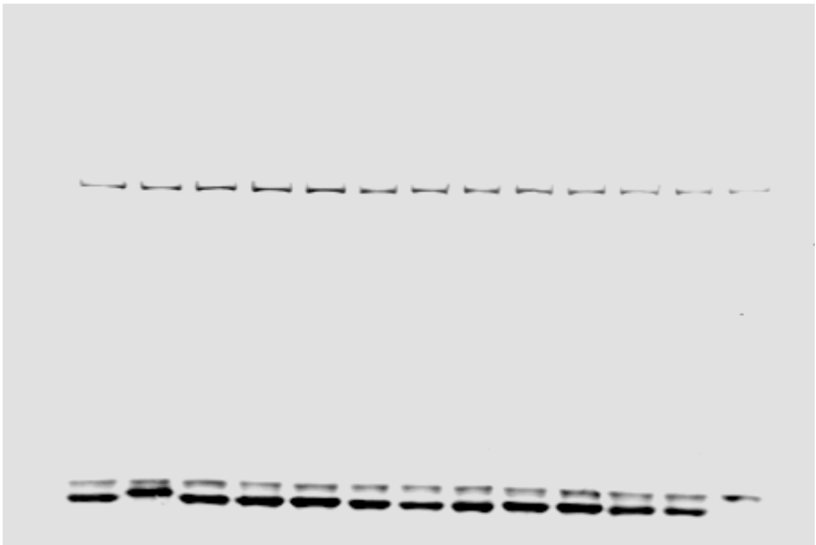

Supplement: Figure 4—source data 1. [file elife-83548-fig4-data1.zip › Figure 4 Source data 1/Fig 4A right lower unlabelled.png]

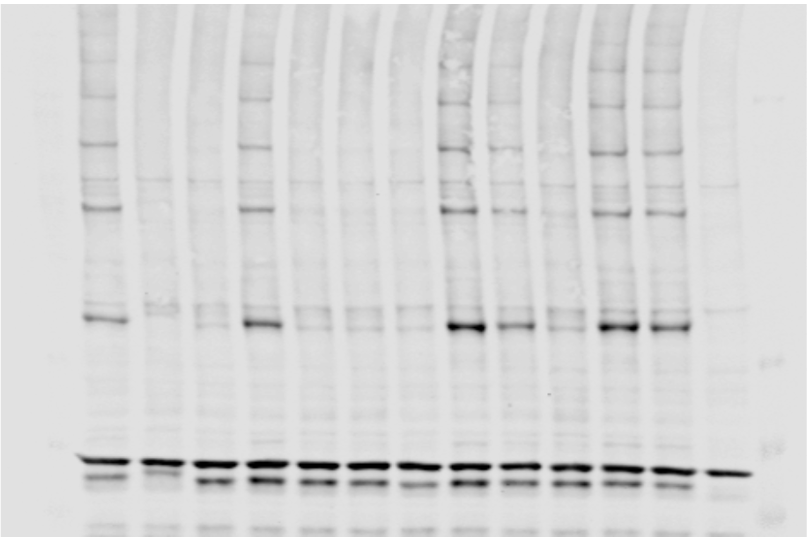

Supplement: Figure 4—source data 1. [file elife-83548-fig4-data1.zip › Figure 4 Source data 1/Fig 4A right upper unlabelled.png]

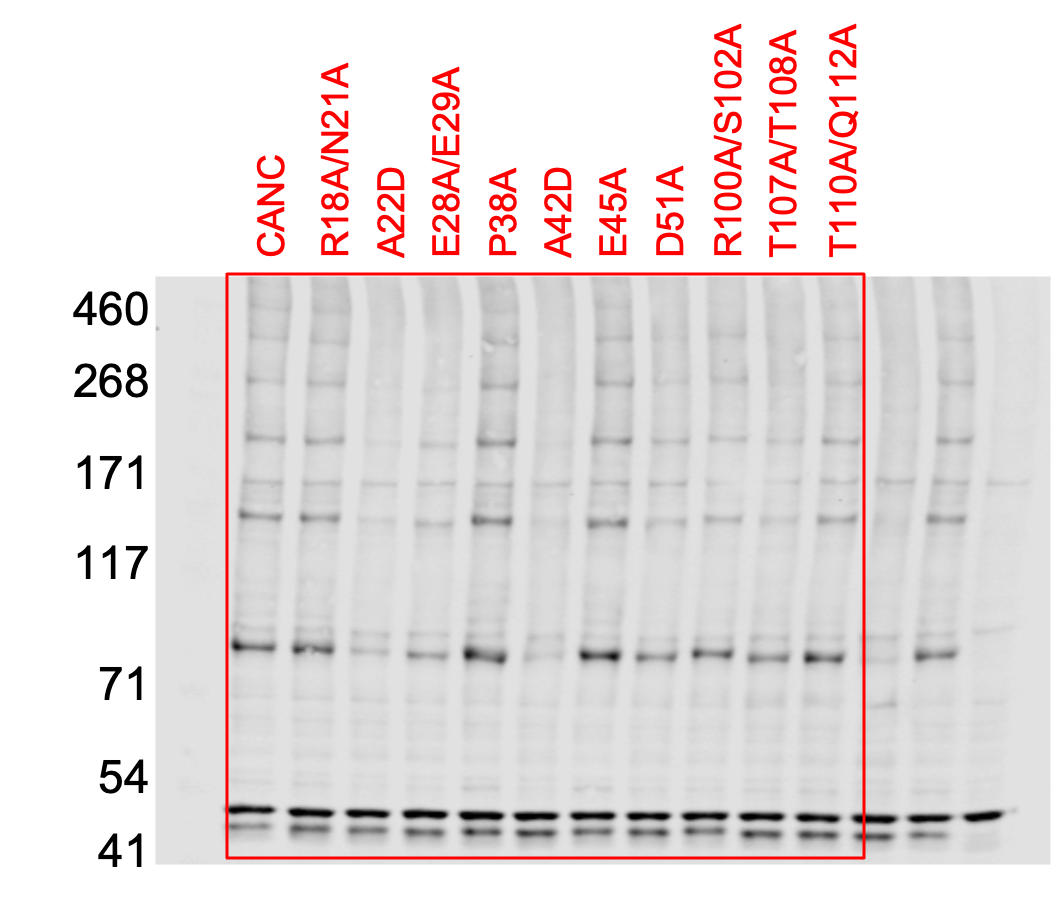

Supplement: Figure 4—source data 1. [file elife-83548-fig4-data1.zip › Figure 4 Source data 1/Fig 4A left upper.png]

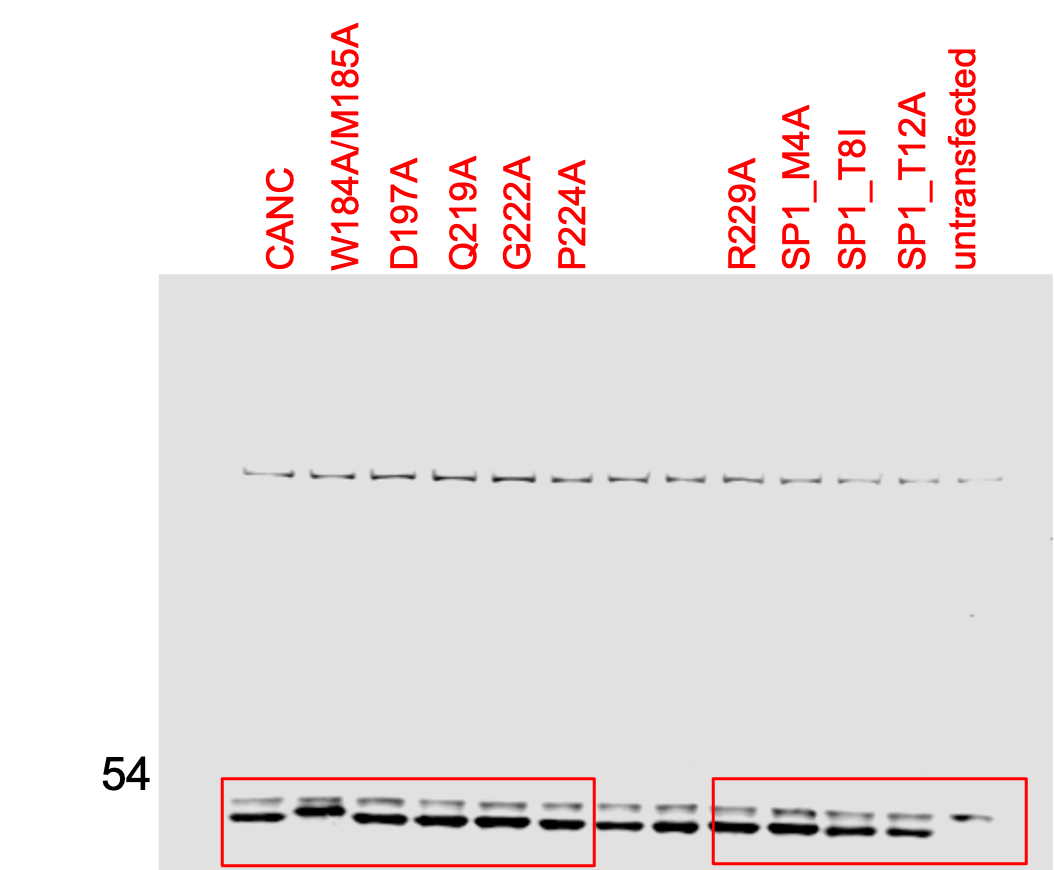

Supplement: Figure 4—source data 1. [file elife-83548-fig4-data1.zip › Figure 4 Source data 1/Fig 4A right lower.png]

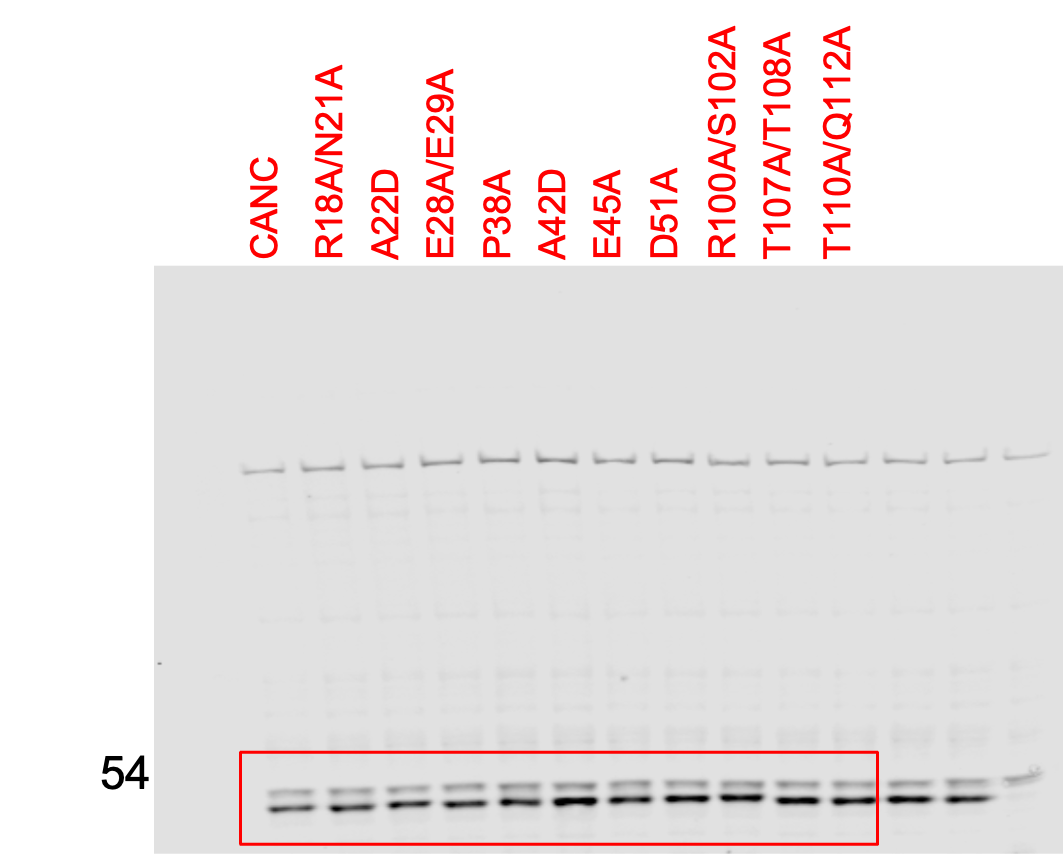

Supplement: Figure 4—source data 1. [file elife-83548-fig4-data1.zip › Figure 4 Source data 1/Fig 4A left lower.png]

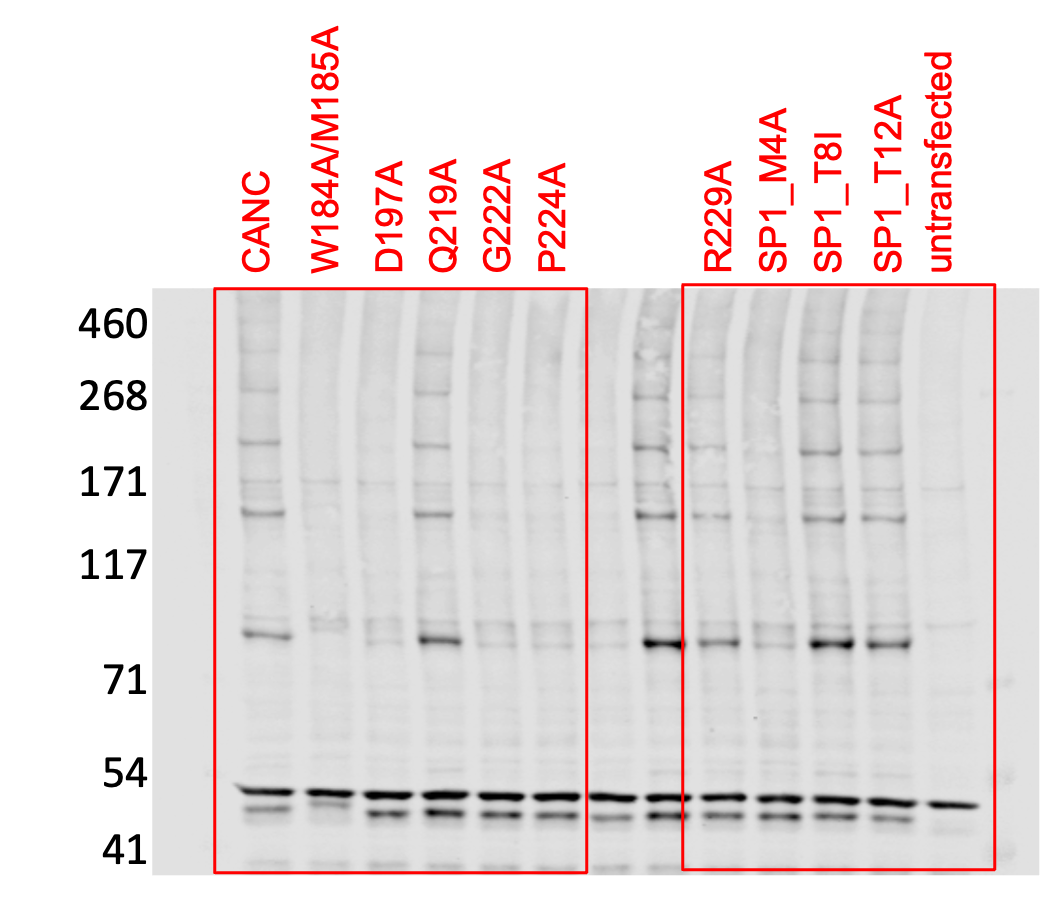

Supplement: Figure 4—source data 1. [file elife-83548-fig4-data1.zip › Figure 4 Source data 1/Fig 4A right upper.png]

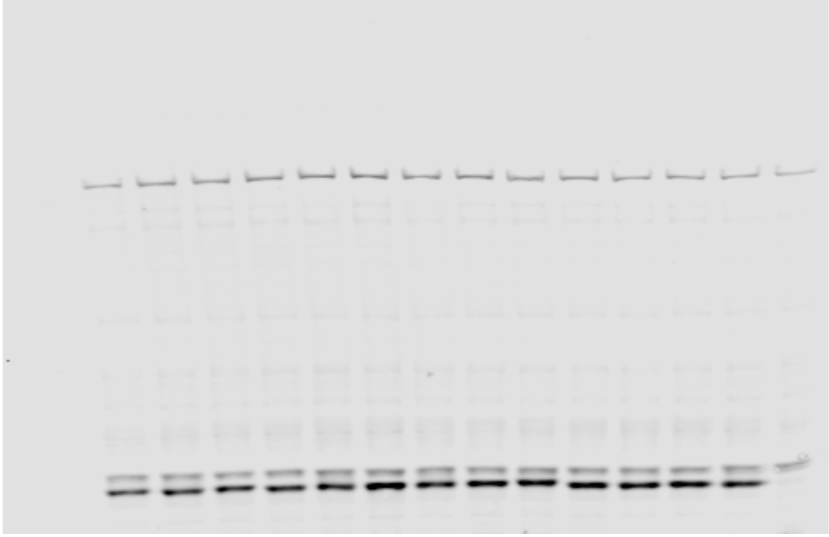

Supplement: Figure 4—source data 1. [file elife-83548-fig4-data1.zip › Figure 4 Source data 1/Fig 4A left lower unlabelled.png]

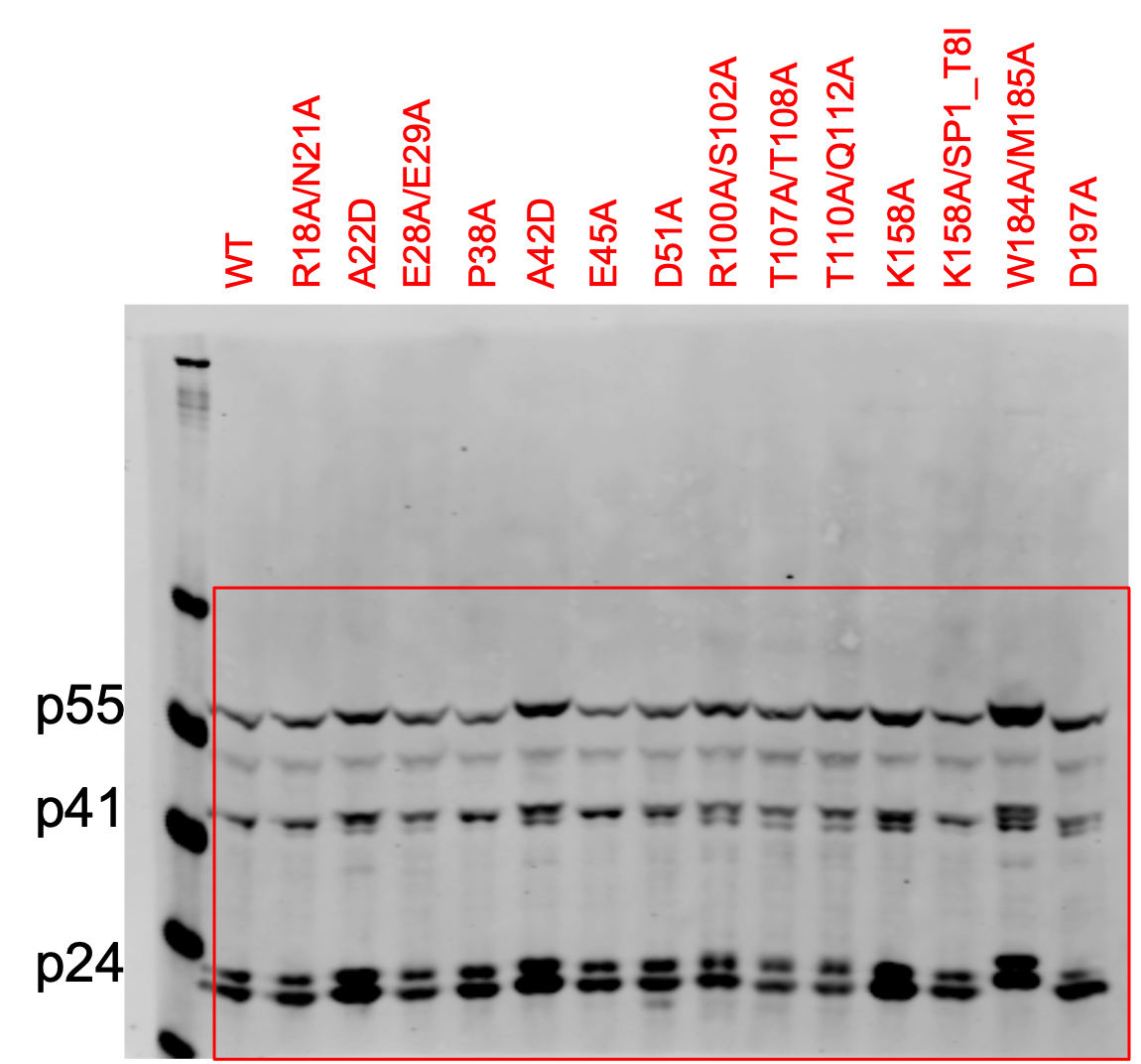

Supplement: Figure 4—figure supplement 2—source data 1. [file elife-83548-fig4-figsupp2-data1.zip › Figure 4-figure supplement 2 source data 1/Figure 4-figure supplement 2-upper left.png]

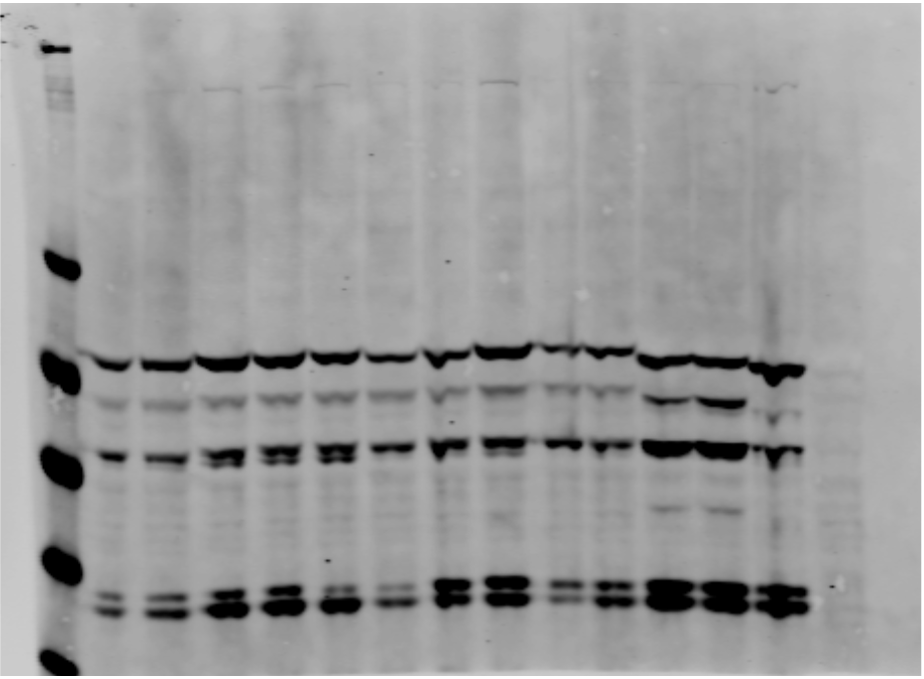

Supplement: Figure 4—figure supplement 2—source data 1. [file elife-83548-fig4-figsupp2-data1.zip › Figure 4-figure supplement 2 source data 1/Figure 4-figure supplement 2-upper right unlabelled.png]

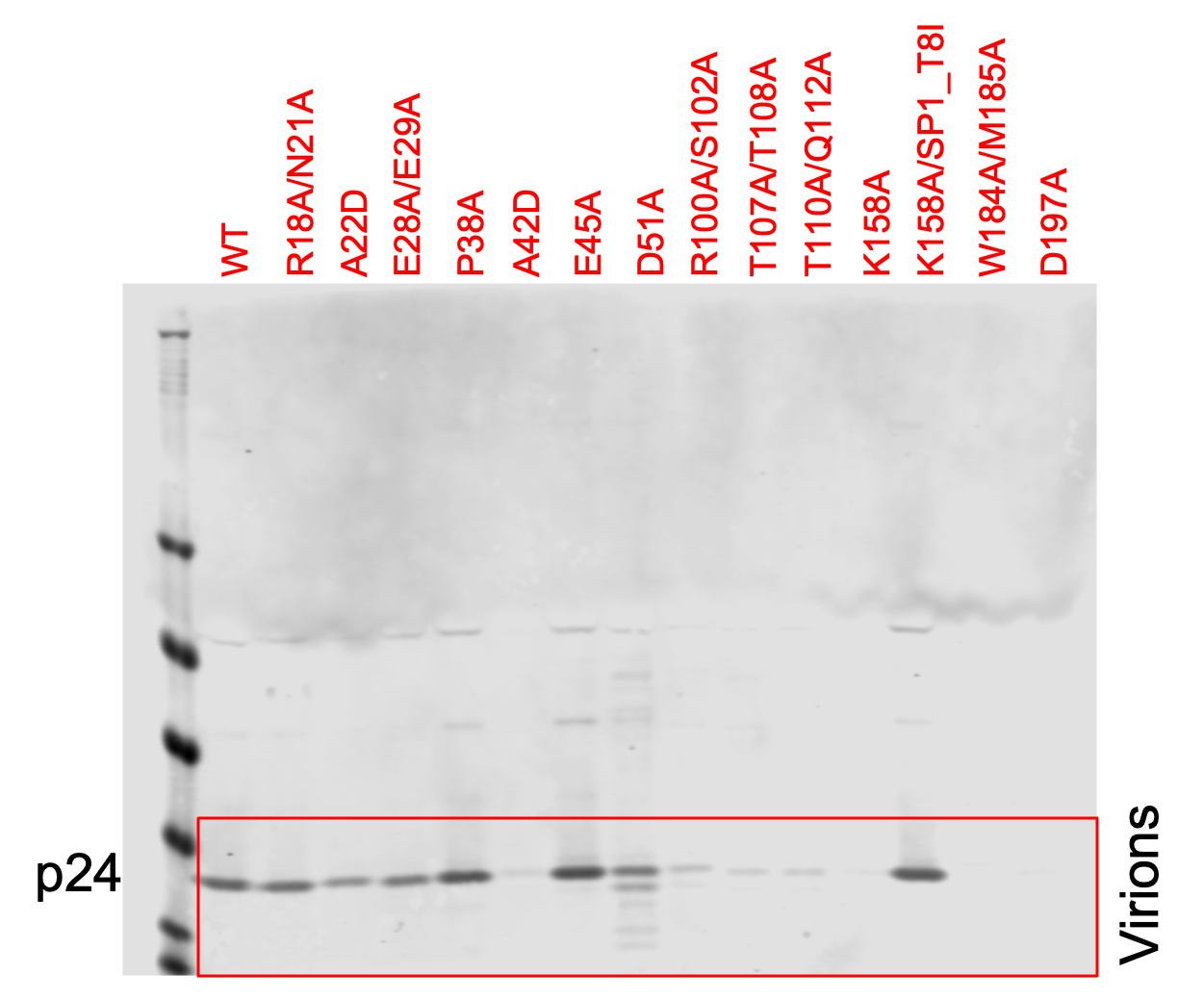

Supplement: Figure 4—figure supplement 2—source data 1. [file elife-83548-fig4-figsupp2-data1.zip › Figure 4-figure supplement 2 source data 1/Figure 4-figure supplement 2-lower left.png]

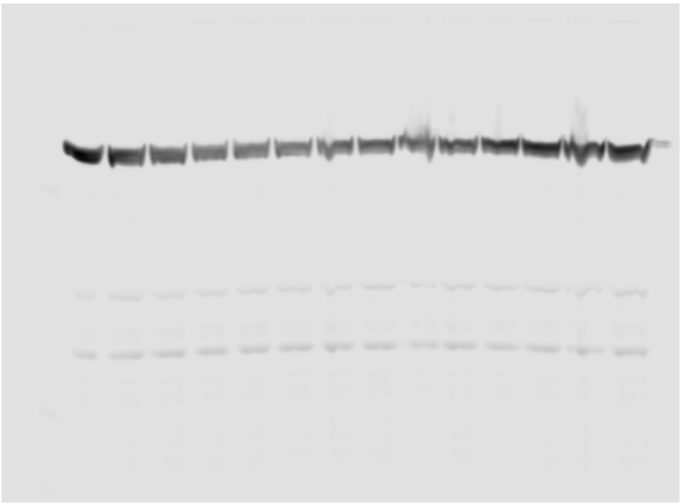

Supplement: Figure 4—figure supplement 2—source data 1. [file elife-83548-fig4-figsupp2-data1.zip › Figure 4-figure supplement 2 source data 1/Figure 4-figure supplement 2-center right unlabelled.png]

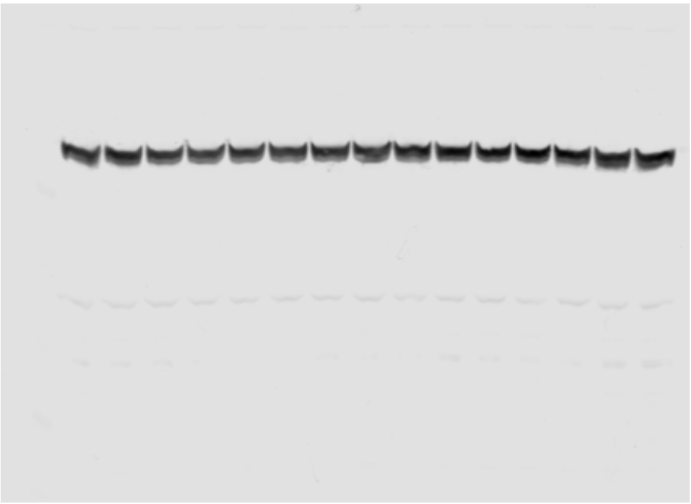

Supplement: Figure 4—figure supplement 2—source data 1. [file elife-83548-fig4-figsupp2-data1.zip › Figure 4-figure supplement 2 source data 1/Figure 4-figure supplement 2-center left unlabelled 6.png]

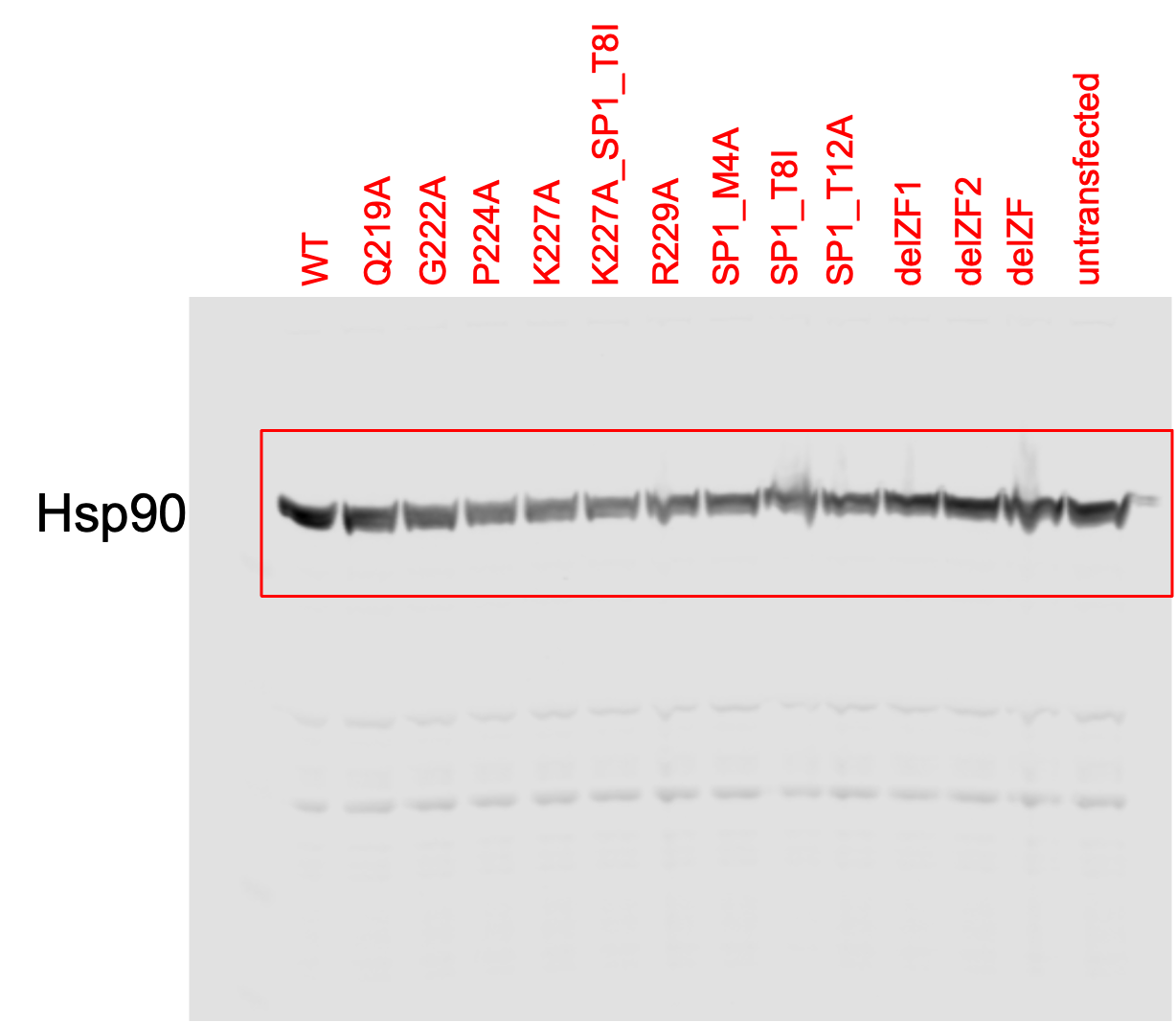

Supplement: Figure 4—figure supplement 2—source data 1. [file elife-83548-fig4-figsupp2-data1.zip › Figure 4-figure supplement 2 source data 1/Figure 4-figure supplement 2-center right.png]

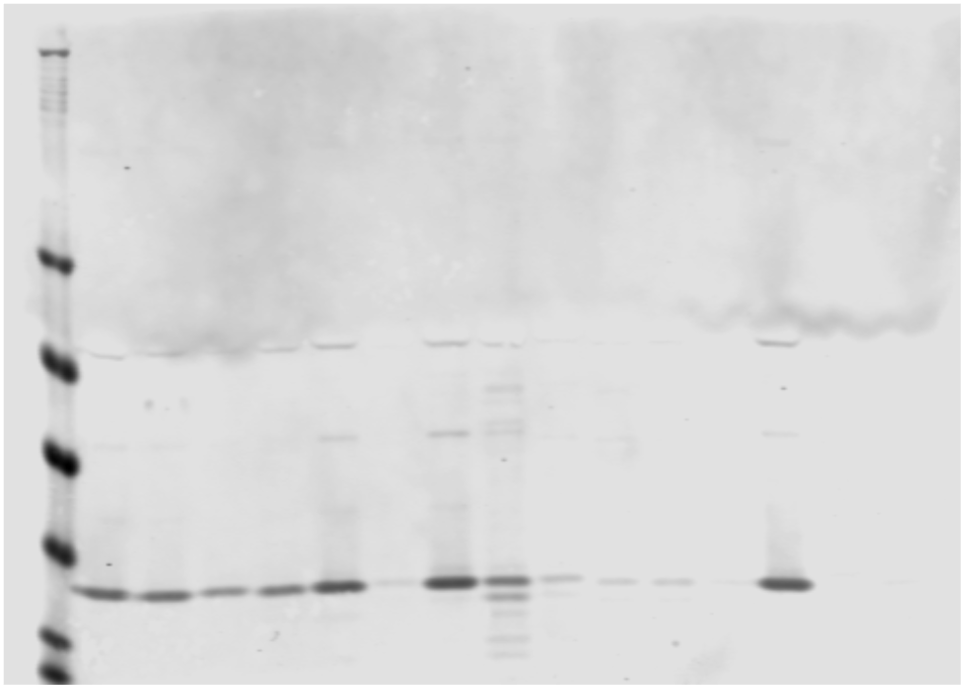

Supplement: Figure 4—figure supplement 2—source data 1. [file elife-83548-fig4-figsupp2-data1.zip › Figure 4-figure supplement 2 source data 1/Figure 4-figure supplement 2-lower left unlabelled.png]

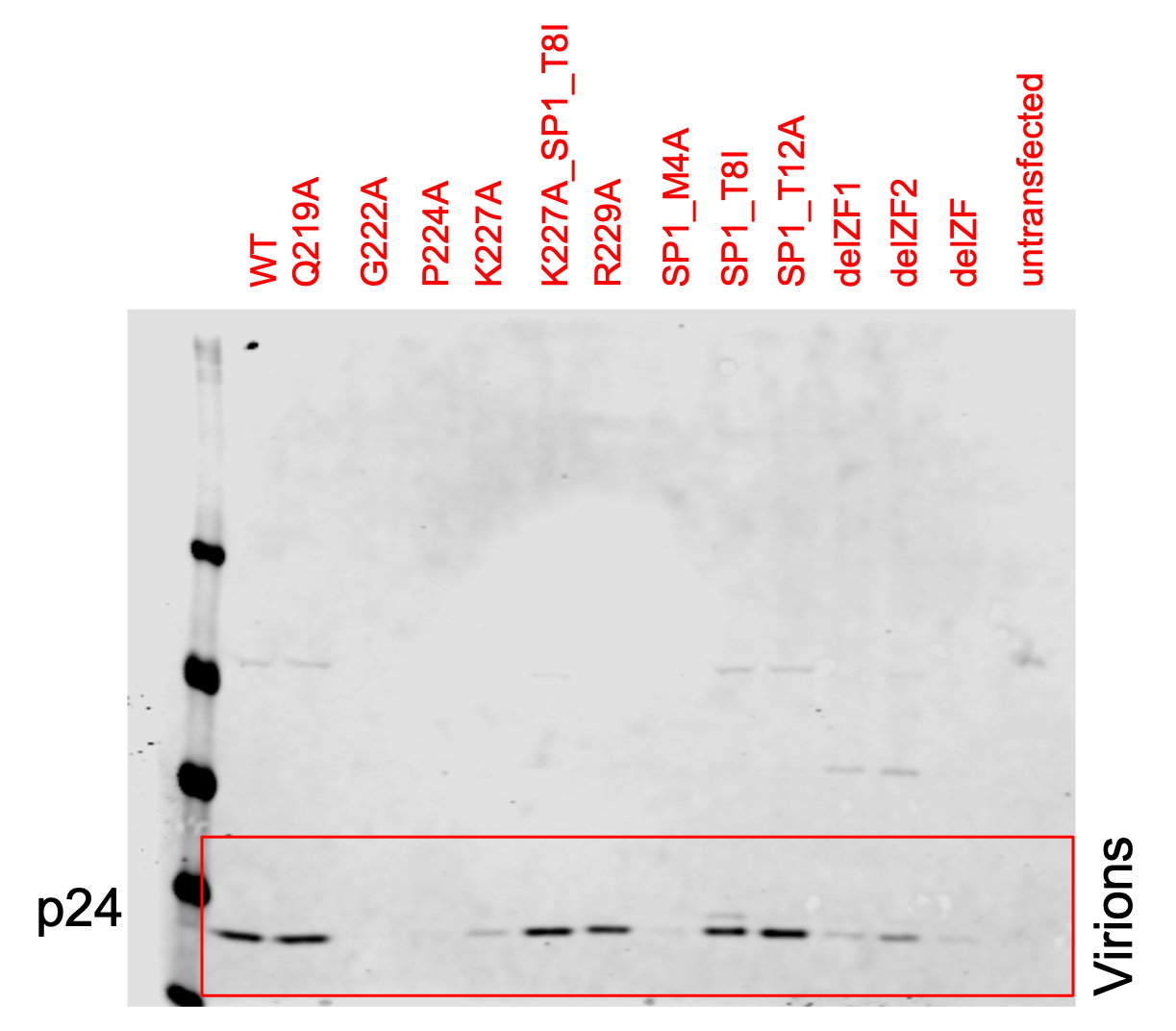

Supplement: Figure 4—figure supplement 2—source data 1. [file elife-83548-fig4-figsupp2-data1.zip › Figure 4-figure supplement 2 source data 1/Figure 4-figure supplement 2-lower right.png]

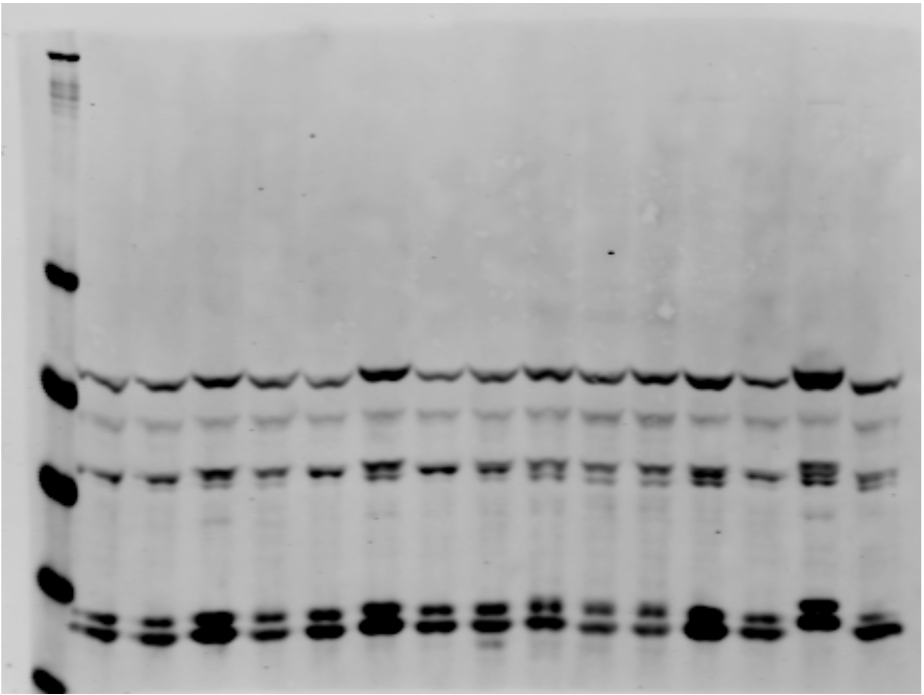

Supplement: Figure 4—figure supplement 2—source data 1. [file elife-83548-fig4-figsupp2-data1.zip › Figure 4-figure supplement 2 source data 1/Figure 4-figure supplement 2-upper left unlabelled.png]

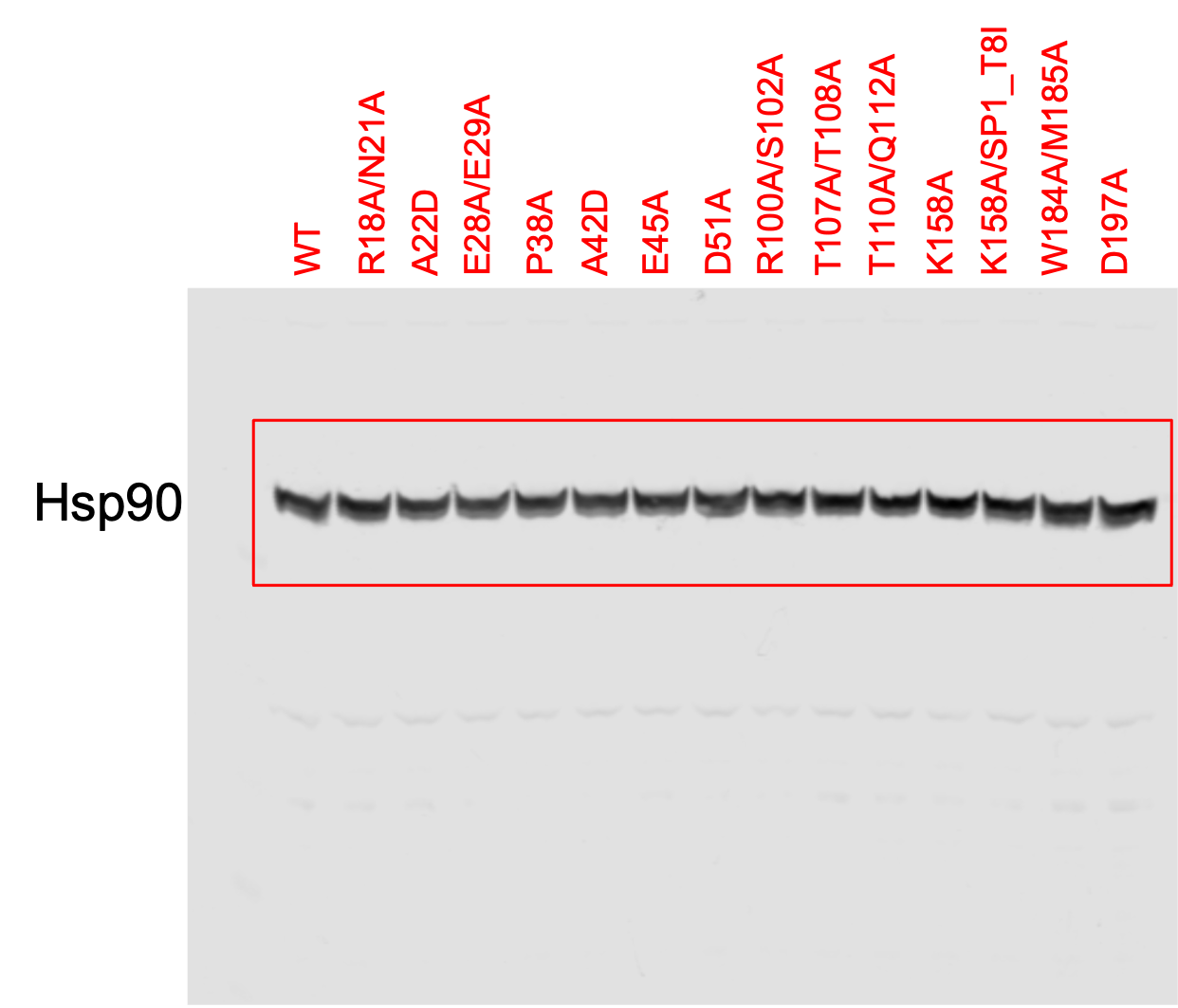

Supplement: Figure 4—figure supplement 2—source data 1. [file elife-83548-fig4-figsupp2-data1.zip › Figure 4-figure supplement 2 source data 1/Figure 4-figure supplement 2-center left.png]

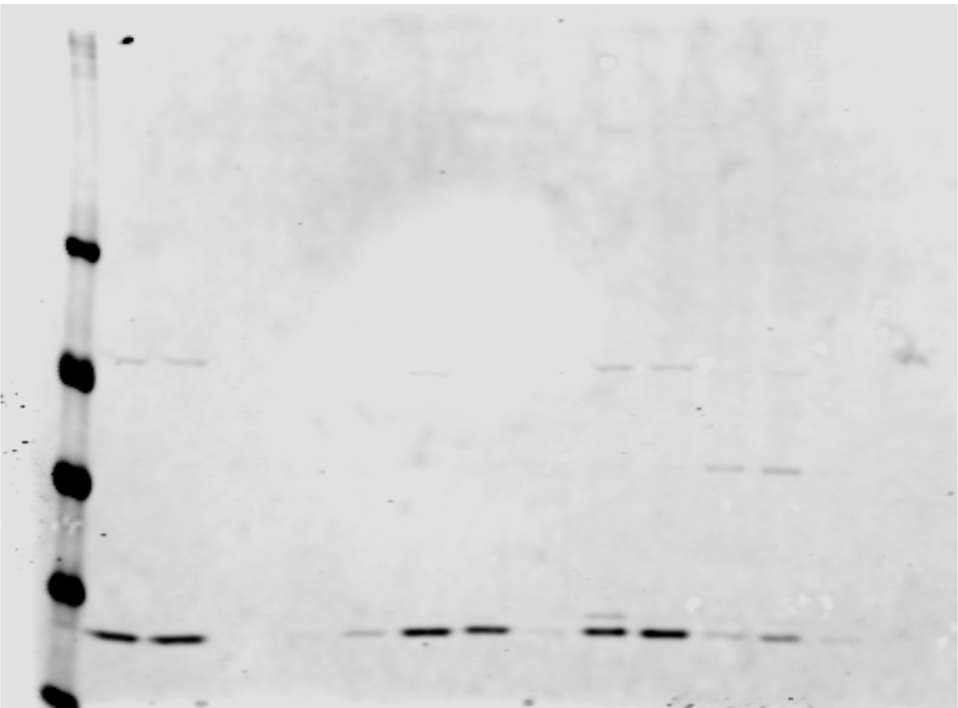

Supplement: Figure 4—figure supplement 2—source data 1. [file elife-83548-fig4-figsupp2-data1.zip › Figure 4-figure supplement 2 source data 1/Figure 4-figure supplement 2-lower right unlabelled.png]

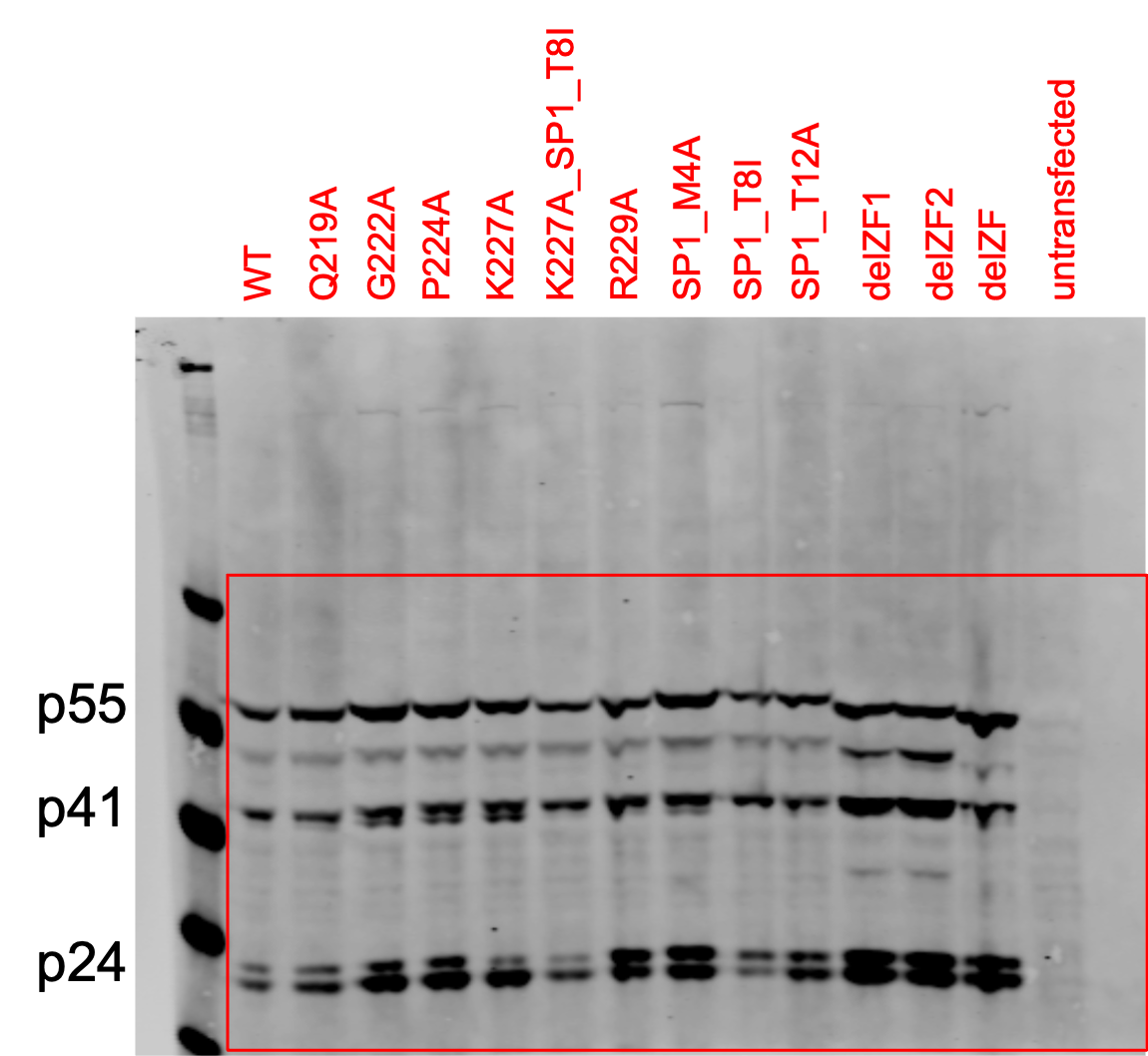

Supplement: Figure 4—figure supplement 2—source data 1. [file elife-83548-fig4-figsupp2-data1.zip › Figure 4-figure supplement 2 source data 1/Figure 4-figure supplement 2-upper right.png]

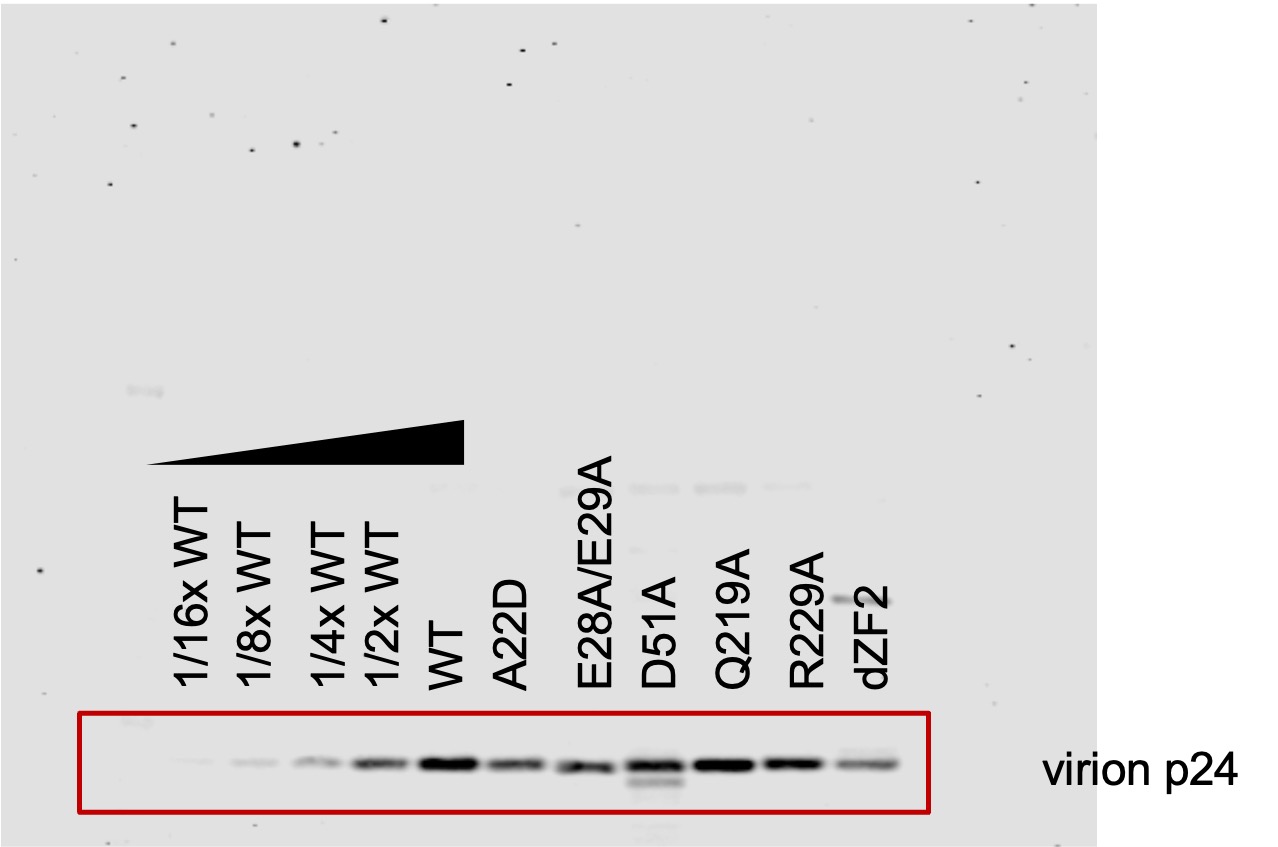

Supplement: Figure 4—figure supplement 4—source data 1. [file elife-83548-fig4-figsupp4-data1.zip › Figure 4-figure supplement 4 Source data 1/Figure 4-figure supplement 4A.jpg]

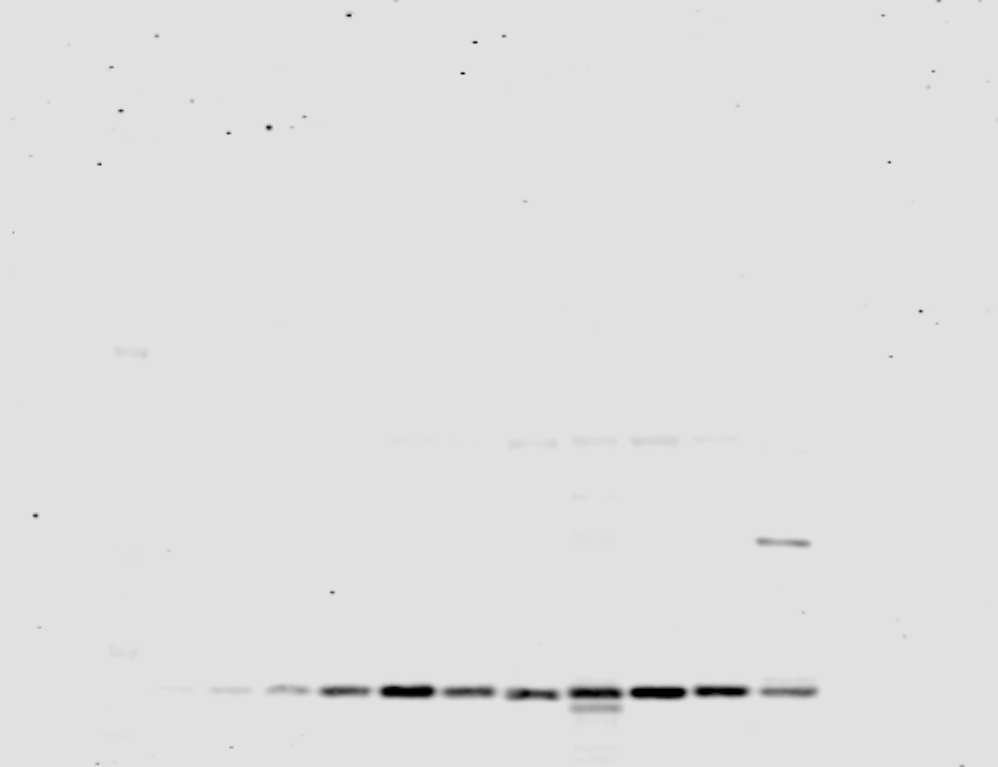

Supplement: Figure 4—figure supplement 4—source data 1. [file elife-83548-fig4-figsupp4-data1.zip › Figure 4-figure supplement 4 Source data 1/Figure 4-figure supplement 4A unlabelled.png]

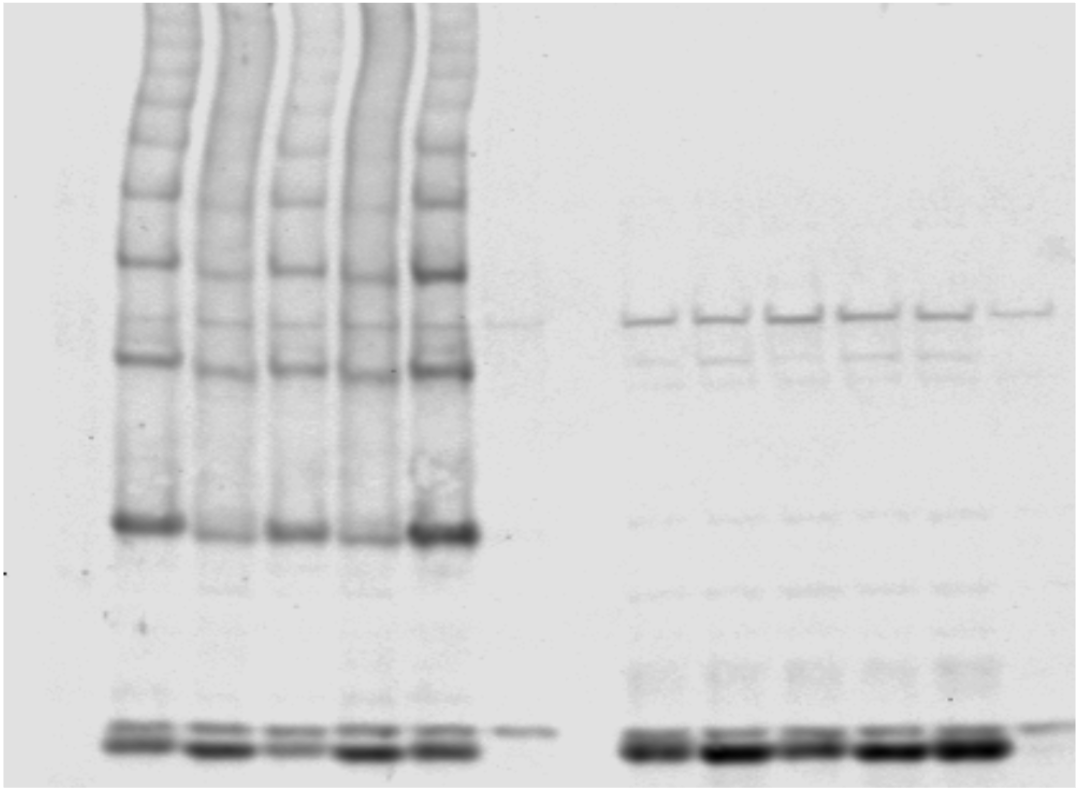

Supplement: Figure 5—source data 1. [file elife-83548-fig5-data1.zip › Figure 5 source data 1/Fig 5A unlabelled.png]

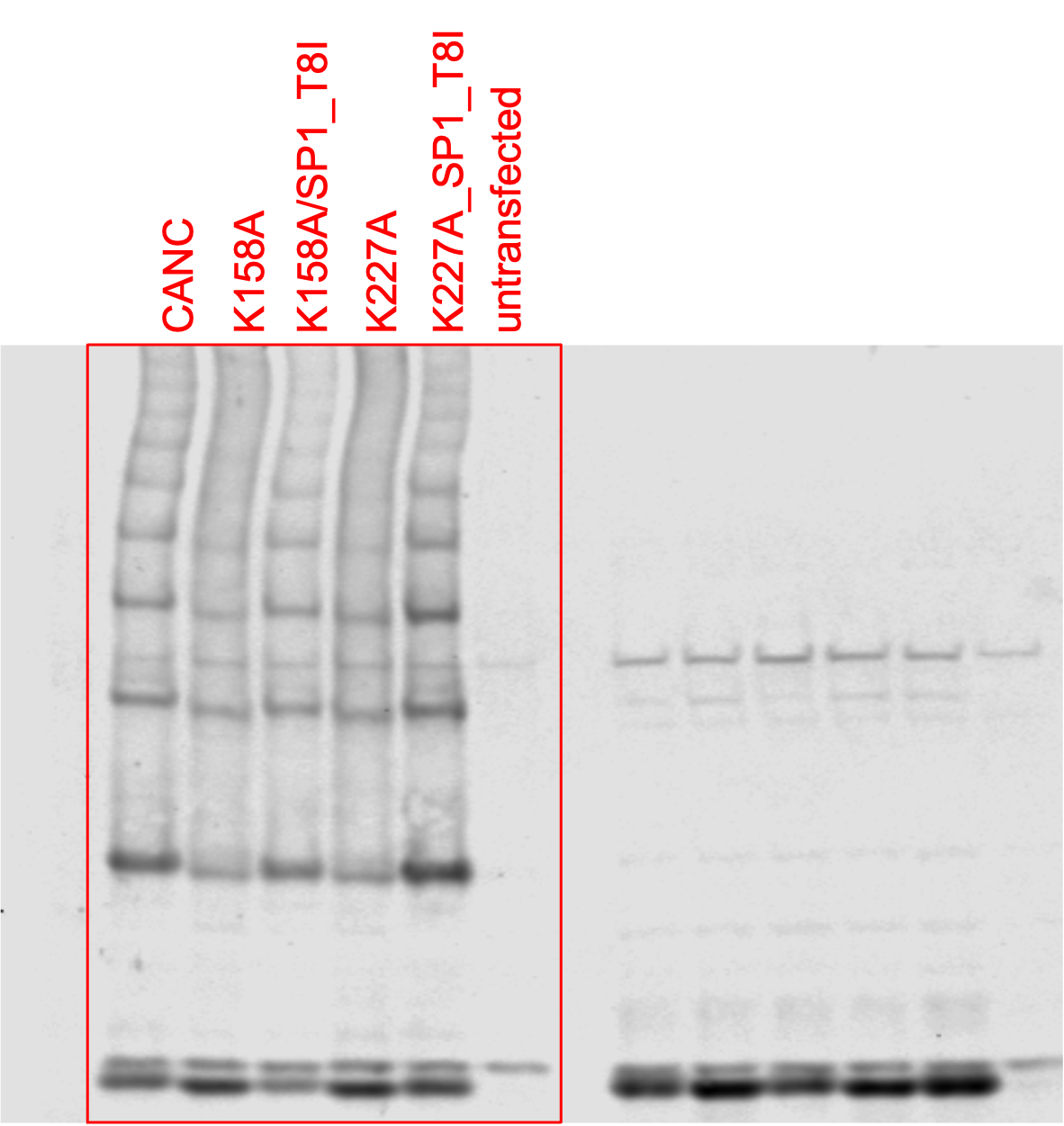

Supplement: Figure 5—source data 1. [file elife-83548-fig5-data1.zip › Figure 5 source data 1/Fig 5A upper.png]

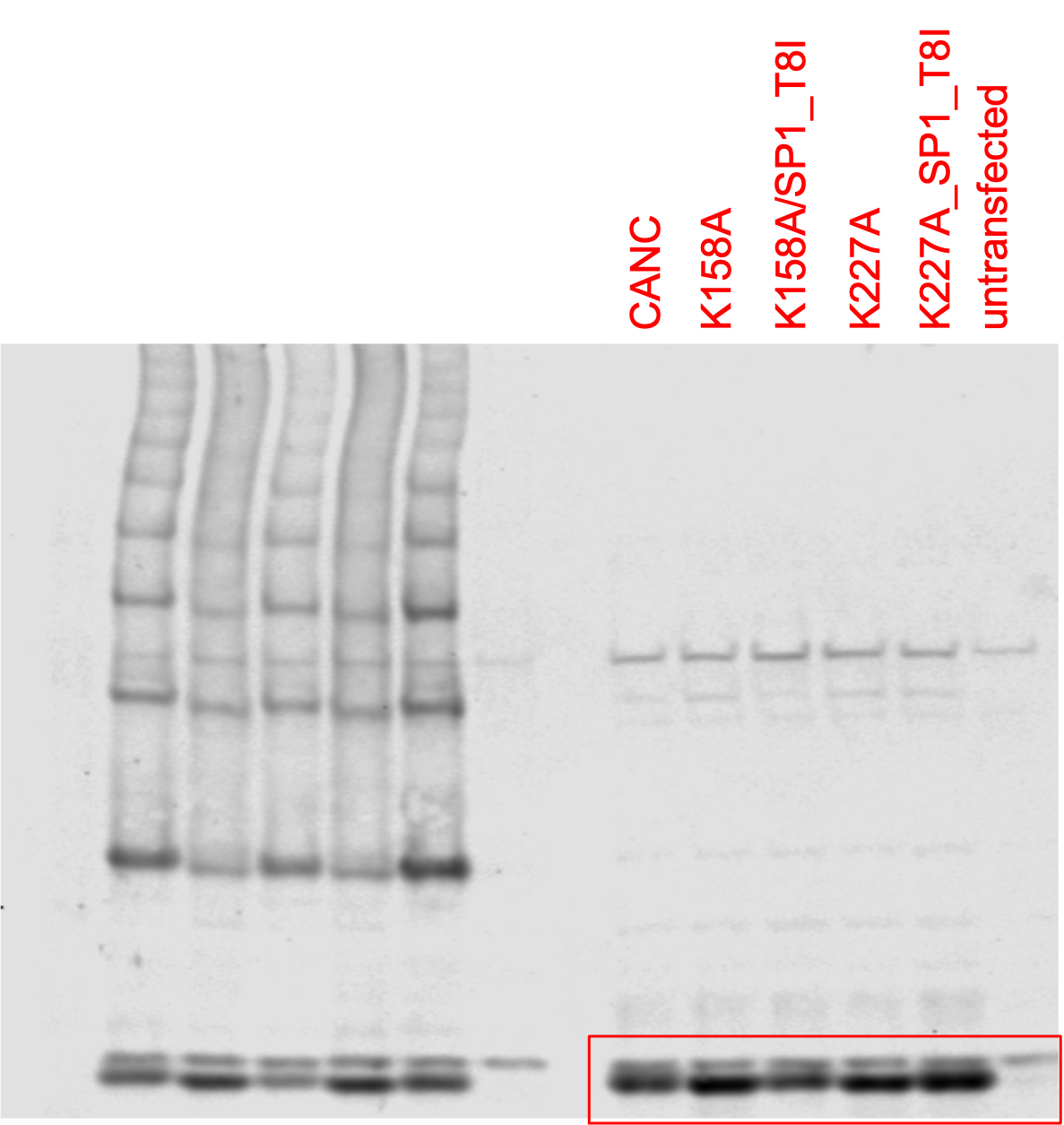

Supplement: Figure 5—source data 1. [file elife-83548-fig5-data1.zip › Figure 5 source data 1/Fig 5A lower.png]
